# A Novel *Polycipiviridae* Virus Identified in *Pteropus lylei* Stools

**DOI:** 10.1128/MRA.01662-18

**Published:** 2019-04-11

**Authors:** Sarah Temmam, Vibol Hul, Thomas Bigot, Thavry Hoem, Christopher Gorman, Veasna Duong, Philippe Dussart, Julien Cappelle, Marc Eloit

**Affiliations:** a Institut Pasteur, Biology of Infection Unit, Inserm U1117, Pathogen Discovery Laboratory, Paris, France; b Virology Unit, Institut Pasteur du Cambodge, Institut Pasteur International Network, Phnom Penh, Cambodia; c Institut Pasteur, Bioinformatics and Biostatistics Hub (C3BI), USR 3756 IP CNRS, Paris, France; d Epidemiology and Public Health Unit, Institut Pasteur du Cambodge, Institut Pasteur International Network, Phnom Penh, Cambodia; e UMR ASTRE, CIRAD, INRA, Université de Montpellier, Montpellier, France; f UMR EpiA, VetAgro Sup, INRA, Marcy l'Etoile, France; g Ecole Nationale Vétérinaire d’Alfort, Université Paris-Est, Maisons-Alfort, France; KU Leuven

## Abstract

*Polycipiviridae* is a recently recognized viral family within the order *Picornavirales* with unusual genome organization and phylogenetic placement. Viruses belonging to this family were only reported from arthropod hosts. We describe here the first full genome of a distant polycipivirus-related virus identified in frugivorous bat stools in Cambodia.

## Abstract (precis)

*Polycipiviridae* is a recently recognized viral family within the order *Picornavirales* with unusual genome organization and phylogenetic placement. Viruses belonging to this family were only reported from arthropod hosts.

## ANNOUNCEMENT

*Picornavirales* consists of nonenveloped viruses characterized by a positive-sense nonsegmented single-stranded RNA (ssRNA) genome and a polyprotein gene expression strategy in which the structural protein module codes for three capsid domains and the nonstructural module codes for the viral helicase and RNA-dependent RNA polymerase (RdRP) (1). Knowledge about picornavirus host range, geographical distribution, and genome organization has exploded due to the democratization of high-throughput sequencing and the identification of novel picorna-like viruses in diverse samples (2). New picornaviruses with a polycistronic genome organization were recently reported in arthropods; *Polycipiviridae* consists of monopartite genomes of 11 kb with four open reading frames (ORFs) in the 5′ region (coding for the structural proteins), followed by an intergenic region and a single ORF coding for the replicase complex (3).

Bats are a major mammalian reservoir of viruses (4). Recent metagenomic studies have highlighted the unexpected diversity of viral communities in bats (5, 6). Bat-associated picornaviruses were reported in *Picornaviridae* (e.g., bat kobivirus, hepatovirus, and mischivirus); *Iflaviridae* (bat iflavirus) and *Dicistroviridae* (bat cripavirus), possibly representing a passive carriage through food; and in unassigned groups (e.g., bat-associated posalivirus, fisalivirus, felisavirus, and dicibavirus) (7, 8). We report here the characterization of the full-genome sequence of the first bat-associated polycipivirus.

A total of 214 *Pteropus lylei* rectal swabs were collected between May 2015 and July 2016 in Kandal Province, Cambodia. Bats were captured using mistnets; handling and sampling were conducted following the FAO guidelines (9). Swabs were pooled and clarified at 10,000 × *g* for 15 min before ultracentrifugation at 100,000 × *g* for 1 h. Total nucleic acids were extracted from the resuspended pellet with the QIAamp cador pathogen mini kit (Qiagen, Courtaboeuf, France) according to the manufacturer’s recommendations, except that carrier RNA was substituted by linear acrylamide (Life Technologies, Courtaboeuf, France). DNA was digested with the Turbo DNase reagent (Ambion, Life Technologies). Total RNA was further purified with the RNeasy cleanup protocol (Qiagen) and used as the template for next-generation sequencing (NGS) library preparation using the SMARTer stranded total RNA-seq kit v2, pico input mammalian (TaKaRa Bio, Saint-Germain-en-Laye, France). Libraries were sequenced in a 2 × 75-bp format on a NextSeq 500 sequencer to produce 45.7 million reads. An in-house bioinformatics pipeline comprised quality check and trimming (AlienTrimmer package [10]), *de novo* assembly (Megahit tool [11]), ORF prediction (https://figshare.com/articles/translateReads_py/7588592), and a sequence search against the protein reference viral database (12; https://rvdb-prot.pasteur.fr), followed by the verification that nothing else but viruses were found as better hits when the sequences were subjected to a BLAST search against the whole NCBI/nonredundant (nr) protein database.

**FIG 1:**
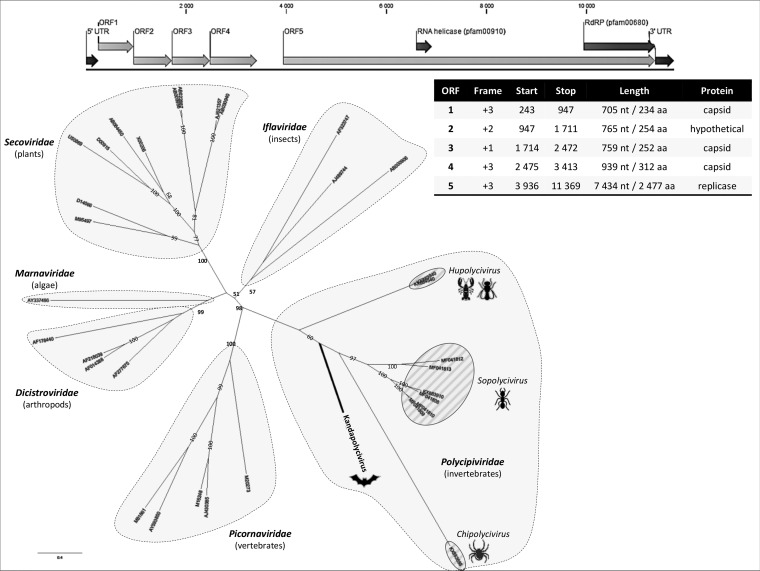
Genome organization of *Kandapolycivirus*, phylogenetic analysis of polycipiviruses, and representative members of the *Picornavirales* order. RNA-dependent RNA polymerase domains were retrieved from Koonin et al. (2) and Olendraite et al. (3) with corresponding accession numbers presented on the tree. Complete amino acid sequences were aligned with MAFFT with the L-INS-I parameter (13). The best amino acid substitution models that fitted the data were determined with ATGC start model selection (14) implemented in PhyML with smart model selection (www.atgc-montpellier.fr/phyml-sms/) using the corrected Akaike information criterion. Phylogenetic trees were constructed using the maximum likelihood (ML) method implemented through the RAxML program under the CIPRES Science Gateway portal (15) according to the selected substitution model. Nodal support was evaluated using the “automatic bootstrap replicates” parameter. Supported nodes (i.e., with bootstrap values above 50) are represented, and bold type indicates nodes defining a family.

A large single contig of 11,745 bp with low amino acid identity to *Polycipiviridae* viruses was obtained. With an average coverage of >3,900× and more than 600,000 reads, this novel virus (tentatively named *Kandapolycivirus*) has a G+C content of 39.25% and the classical genome organization of polycipiviruses, namely, four ORFs in the 5′ part of the genome, among which ORF1 (234 amino acids [aa]), ORF3 (252 aa), and ORF4 (312 aa) code for capsid-like proteins, and a large ORF (ORF5; 2,477 aa) in the 3′-coding region for the replicase module, with RNA helicase and RdRP domains (Fig. 1). ORF2 codes for a protein of 255 aa of unknown function but has several *O*-glycosylation sites, possibly constituting the fourth capsid ORF that is characteristic of *Polycipiviridae*. Phylogenetic analyses performed on the complete RdRP domain of polycipiviruses and representative *Picornavirales* viruses places *Kandapolycivirus* in the *Polycipiviridae* clade (Fig. 1). Interestingly, *Kandapolycivirus* locates in a distinct putative genus from the *Chipolycivirus* (arachnid-associated viruses), the *Sopolycivirus* (ant-specific viruses), and the *Hupolycivirus* (crustacean- and insect-associated viruses) genera.

### Data availability.

The genome sequence of *Kandapolycivirus* was deposited in GenBank under accession number MK161350. Raw data corresponding to the *Kandapolycivirus* genome were deposited into the NCBI SRA database under the accession number PRJNA516387.
